# Effectiveness and safety of chemotherapy combined with immunomodulatory therapies for multiple myeloma: A protocol for systematic review and meta-analysis

**DOI:** 10.1097/MD.0000000000029093

**Published:** 2022-04-01

**Authors:** Jie Deng, Li Liu, Jianhai Sun, Yanlin Ma, Li Li

**Affiliations:** Department of Oncology, Hubei No. 3 People's Hospital of Jianghan University, Wuhan, Hubei, China.

**Keywords:** chemotherapy, effectiveness, immunomodulatory, multiple myeloma, safety

## Abstract

**Background:**

Multiple myeloma (MM) is considered one of the prevalent malignant plasma cell diseases affecting people. In essence, maintenance treatment is valuable for prolonging the survival time of patients experiencing MM. The majority of the currently used treatment protocols for MM are founded on a combination of chemotherapy and immunomodulatory drugs, of which immunomodulatory drugs seems to be one of the most active drugs. However, in the literature, chemotherapy combined with immunomodulatory therapies have not been unambiguously proven. To systematically appraise and synthesize these results, the present investigation will evaluate whether combining chemotherapy with immunomodulatory therapies an effective and safe approach to treating patients with MM.

**Methods:**

Two authors relied in 7 different databases: PubMed, EMBASE, Cochrane Library, Web of Science, WanFang Database, Chinese Biomedical Literature Database, China National Knowledge Infrastructure and for studies on chemotherapy's effectiveness when combined with immunomodulatory therapies. The authors only considered studies published up to December 16, 2021 and only those written in English or Chinese. They will also carry out selection of studies, extraction of data, along with assessing risk of bias. Besides, they will also use RevMan V.5.3 to conduct data synthesis. They will establish heterogeneity using the *I*^2^ test. At the same time, the authors will evaluate publication bias by making a funnel plot and conducting the Begg as well as Egger tests.

**Ethics and dissemination:**

The present study will not necessitate ethics approval since it will be funded on already published works.

**OSF registration number:**

10.17605/OSF.IO/X7DE4.

## 1 Introduction

Multiple myeloma (MM) is having been considered a prevalent malignant plasma cells condition, accounting for an estimated 10% of all malignancies associated with hematologic diseases and mainly in older adults. In particular, 65 years is considered the median age of diagnosing this disease, with approximately 1% accounting for people younger than the age of 65.^[1]^ Besides, the primary resident in the bone marrow are malignant plasma cells. In others, they can be noticeable in the peripheral blood, as well as other extramedullary locations.^[2]^ Accordingly, most patient experiencing MM denote features such secreting monoclonal immunoglobulin protein, which is generated by the nonstandard plasma cells. Nevertheless, a large pool of myeloma cells tend to secrete only monoclonal, which are uninhibited light chains in 15% to 20% of patients, as well as no monoclonal protein in only <3% of patients.^[1,3]^ To this end, the clinical indicators of MM can be said to be propelled by monoclonal protein, in which malignant cells or cytokines that have been secreted by the malignant cells demonstrate signs of damage to the end-organ: they include hypercalcemia, anemia, insufficiency of renal, along with illnesses characterized by lytic lesions or compulsive ruptures, jointly referred to as CRAB characteristics.^[4]^

Maintenance treatment after transplantation of autologous stem cell or, in some cases, remission, is crucial for eliminating the minimal residual disease, which contributes to a prolonged survival in patients experiencing MM.^[5,6]^ Presently, the main drugs used in the maintenance treatment are “glucocorticoid, interferon, immunomodulatory drugs (IMiDs), and proteasome inhibitors”, among others. In particular, continued utilization of some of these medicines is restricted by serious adversative consequences, including change in mood, myalgia, infection, along with toxicity of hematological.^[7]^ Accordingly, IMiDs can specifically deal with myeloma cells as well as bone marrow hematopoietic microenvironment. Thus, they play two-fold activities, including to kill tumor cells as well as helping regulation of immune. At the same time, maintenance therapies that are founded on thalidomide and lenalidomide, among other IMiDs, has the capacity of substantially causing prolonging of the progression-free survival. However, it is still constentious whether it can lead to enhancing overall survival. Although chemotherapies have been utilized broadly over the years, there are still no known role of this intervention when combined immunomodulatory therapies.^[8,9]^ Therefore, the present study intends to investigate whether chemotherapy combined with immunomodulatory therapies for MM will be effective and safe.

## 2 Objective

The study aims at conducting a systematic review of randomized controlled trials (RCTs) to investigate the comparative effectiveness and safety of chemotherapy combined with immunomodulatory therapies for MM.

## 3 Methods and analysis

We will set the preferred reporting items for systematic reviews and meta-analyses protocols as a guide book for the protocol.^[10]^ This systematic review protocol has been registered with the Open Science Framework (10.17605/OSF.IO/X7DE4).

## 4 Eligibility criteria

### 4.1 Types of studies

RCTs comparing the effectiveness of treatment, including chemotherapy combined with immunomodulatory therapies with treatment including chemotherapy alone, IMiDs alone, another treatment, or no treatment in the treatment of MM.

### 4.2 Types of participants

Participants were made up of both patients who had been newly diagnosed as well as those already treated of their MM.

### 4.3 Types of interventions

Treatment including chemotherapy combined with immunomodulatory therapies versus treatment with chemotherapy alone, IMiDs alone, another intervention, or no intervention.

### 4.4 Types of outcome measures

The primary outcomes are overall survival, progression-free survival, response rate (defined as either classical response rates. The secondary outcomes are toxicities and quality of life.

## 5 Search methods for identification of studies

We will use 2 authors to search 7 specific databases: PubMed, EMBASE, Cochrane Library, Web of Science, WanFang Database, Chinese Biomedical Literature Database, China National Knowledge Infrastructure and for studies on chemotherapy's efficacy and safety when it is combined with immunomodulatory therapies. The authors will consider articles published until December 16, 2021, and only those that are written in either English or Chinese languages. Additionally, the authors will search grey literature and carry out hand-searching of reference lists on all studies included, pertinent reviews, along with pertinent documents. The authors will base their search on Medical Subject Headings, which entails a wide range of terms and keywords, including “myeloma”, “immunomodulatory”, and “chemotherapy”.

## 6 Data collection and analysis

### 6.1 Study identification

The study will employ the utilization of EndNote V.9.0 software in collating search results and performing filtering of the results. Two independent authors will remove all the duplicates and then review all titles and abstracts of the studies identified to exclude irrelevant sections. Accordingly, after they obtain the full articles, they will re-examine them for more details. In case of any conflict during this exercise, a third author will come in to resolve the disagreement.

### 6.2 Data extraction and management

The authors planned on following the process of extracting data as per suitable trials. They will first consider the articles’ publication date, researches’ details such as patients’ features, sample size, and adversative events, among others. Besides, 2 authors conduct this process independently using the standard table of extracting data. In case of any conflict during this exercise, a third author will come in to resolve the disagreement.

### 6.3 Assessment of risk of bias in included studies

Two independent authors will evaluate the standards of the literature to be included in the study by means of the Cochrane Collaboration bias risk assessment tool. The evaluation will include processes such as generating of random sequences, blinding, allocating concealments, selecting outcome reporting procedure, and so on. Accordingly, the pertinent Cochrane Intervention System Evaluation Manual will distinguish risks and low, high, or unclear.

### 6.4 Measures of treatment effect

The risk ratio with 95% confidence intervals (CIs) will be employed in analyzing dichotomous data. However, the mean difference with 95% CIs will be utilized in analyzing continuous data. Similarly, the standardized mean differences with 95% CIs will be employed where there are various scales utilized in measuring some specific outcome variables. Overall, where the significant heterogeneity will be detected, the author will use the random-effects model.

### 6.5 Assessment of heterogeneity

To test or evaluate heterogeneity, the authors will utilize the *I*^*2*^. Where the outcome of *I*^*2*^ is found to be over 50%, the authors will consider that there is a substantial heterogeneity. However, if the authors manage a descriptive statistical analysis for data synthesis and detecting the latent factors through the use of the subgroup analysis. Similarly, where the *I*^*2*^ will be found to be under 50%, it will deduce that the heterogeneity is low; thus, the *Chi-squared* test will be employed in searching for a statistical heterogeneity.

### 6.6 Assessment of reporting biases

Where more than 10 studies have been included, the funnel plots will be utilized in detecting potential reporting biases. Accordingly, the authors will evaluate publication bias by establishing a funnel plot and conducting the Begg as well as Egger tests.

### 6.7 Sensitivity analysis

The authors will further evaluate the pooled results’ robustness by performing sensitivity analysis, by including only RCTs with a low or moderate risks of bias as well as utilizing an alternative effect model (random effects model vs fixed-effect model).

## 7 Discussion

We believe that the present systematic review will act as the first in assessing the efficacy and safety of combining chemotherapy and immunomodulatory therapies in treating MM. The results of this study demonstrates that there is a gap in literature regarding this topic. Accordingly, the review will be divided into 4 distinct parts, including identifying, inclusion of study, extracting data, and synthesis of data. In exploring effectiveness and safety, it is crucial to systematically combine existing research findings, specifically those aimed at studying its impacts on MM survival outcomes using a systematic evaluation as well as meta-analysis. This integration is likely to establish a guidance that will be critical to select treatment options for clinical maintenance.

## Author contributions

**Conceptualization:** Jie Deng, Li Liu.

**Data curation:** Jie Deng, Li Liu, Jianhai Sun, Yanlin Ma, Li Li.

**Formal analysis:** Jie Deng, Li Liu.

**Funding acquisition:** Li Li.

**Investigation:** Jie Deng, Li Liu, Jianhai Sun, Yanlin Ma.

**Methodology:** Li Li.

**Project administration:** Jie Deng, Li Liu, Jianhai Sun, Li Li.

**Resources:** Li Li.

**Software:** Jie Deng, Li Liu, Jianhai Sun, Yanlin Ma.

**Supervision:** Jie Deng, Jianhai Sun, Yanlin Ma.

**Validation:** Jie Deng, Li Liu.

**Visualization:** Jianhai Sun.

**Writing – original draft:** Jie Deng, Li Li.

**Writing – review & editing:** Li Li.
